# Machine learning approach for the detection of vitamin D level: a comparative study

**DOI:** 10.1186/s12911-023-02323-z

**Published:** 2023-10-16

**Authors:** Nuriye Sancar, Sahar S. Tabrizi

**Affiliations:** 1 Department of Mathematics, Near East University, Nicosia, 99138 Turkey; 2 Department of Computer Engineering, Faculty of Electrical and Computer Engineering, University of Tabriz, Tabriz, Iran

**Keywords:** Machine learning models, Vitamin D, Metabolic syndrome, Comparative study, Multicollinearity, Classification algorithm

## Abstract

**Background:**

After the World Health Organization declared the COVID-19 pandemic, the role of Vitamin D has become even more critical for people worldwide. The most accurate way to define vitamin D level is 25-hydroxy vitamin D(25-OH-D) blood test. However, this blood test is not always feasible. Most data sets used in health science research usually contain highly correlated features, which is referred to as multicollinearity problem. This problem can lead to misleading results and overfitting problems in the ML training process. Therefore, the proposed study aims to determine a clinically acceptable ML model for the detection of the vitamin D status of the North Cyprus adult participants accurately, without the need to determine 25-OH-D level, taking into account the multicollinearity problem.

**Method:**

The study was conducted with 481 observations who applied voluntarily to Internal Medicine Department at NEU Hospital. The classification performance of four conventional supervised ML models, namely, Ordinal logistic regression(OLR), Elastic-net ordinal regression(ENOR), Support Vector Machine(SVM), and Random Forest (RF) was compared. The comparative analysis is performed regarding the model's sensitivity to the participant’s metabolic syndrome(MtS)'positive status, hyper-parameter tuning, sensitivities to the size of training data, and the classification performance of the models.

**Results:**

Due to the presence of multicollinearity, the findings showed that the performance of the SVM(RBF) is obviously negatively affected when the test is examined. Moreover, it can be obviously detected that RF is more robust than other models when the variations in the size of training data are examined. This experiment's result showed that the selected RF and ENOR showed better performances than the other two models when the size of training samples was reduced. Since the multicollinearity is more severe in the small samples, it can be concluded that RF and ENOR are not affected by the presence of the multicollinearity problem. The comparative analysis revealed that the RF classifier performed better and was more robust than the other proposed models in terms of accuracy (0.94), specificity (0.96), sensitivity or recall (0.94), precision (0.95), F1-score (0.95), and Cohen's kappa (0.90).

**Conclusion:**

It is evident that the RF achieved better than the SVM(RBF), ENOR, and OLR. These comparison findings will be applied to develop a Vitamin D level intelligent detection system for being used in routine clinical, biochemical tests, and lifestyle characteristics of individuals to decrease the cost and time of vitamin D level detection.

## Background

The modern history of Vitamin D importance among physicians and scholars began in the mid of 1800s. After the World Health Organization declared the COVID-19 pandemic in March 2020 [1], the importance of Vitamin D had driven the attention of all people worldwide, regardless of their education and professions. Thus, people are more aware of the level of this vital factor in their clinical laboratory test results than before. Vitamin D plays an essential role in our lives. Vitamin D acts as an immune modulator, keeping inflammation at bay while supporting the functions of B cells and T cells, which are essential for tackling infections and forming immunological memory. Vitamin D is a hormone known as a fat-soluble vitamin. In the medical language, it is one of the types of vitamins called Calciferol, soluble in fat and stored in the liver and adipose tissue. It is the only vitamin that can be synthesized in the human body. Vitamin D, taken from the sun and food, is transformed into a more effective chemical by changing the liver and kidney. This kind of vitamin affects the body's calcium homeostasis and bone metabolism [2, 3]. The role of Vitamin D in regulating many cell functions apart from bone mineral metabolism has been studied intensively in recent years [4]. Vitamin D type is divided into two types, namely "Type D2" and "Type D3". Its primary source in the body is that it is synthesized in the skin after exposure to sunlight (Vitamin Type D3). Still, it is also exogenously taken in the diet (Vitamin Type D3 and Vitamin Type D2) [5].

Vitamin D deficiency is a common health problem worldwide. In the 1960s, for the first time, two scholars, namely Whistler and Glisson, described Vitamin D deficiency [6, 7]. This deficiency affects all age groups and causes emerging essential health problems that may lead to many diseases. In addition to healthy bone development, previous studies showed that Vitamin D deficiency increases the incidence of autoimmune diseases [8–10]. Long-term vitamin D insufficiency can have a variety of negative effects on health, including compromised immune function, increased risk of cancer, diabetes mellitus, and cardiovascular conditions [11–15]. Long-term vitamin D deficiency can also result in secondary hyperparathyroidism, bone loss that can cause osteoporosis and fractures, mineralization problems that may eventually cause osteomalacia, and muscular weakness that can result in falls and fractures [16]. Additionally, vitamin D has a significant role in the clinical development of infectious and other acute disorders, including respiratory bacterial infections, tuberculosis, and viral infections [15]. Studies associate Vitamin D deficiency with more severe COVID-19 infection [17, 18]. Besides, a study found significant rough correlations between vitamin D levels and the number of COVID-19 cases especially the death rate caused by this infection [18]. Nowadays, lifestyle conditions of modern life, such as working indoors, not performing outdoor activities adequately, and malnutrition, could increase the risk of Vitamin D deficiency [19].

Serum 25-Hydroxy Vitamin D (25-OH-D) measurement is usually performed to evaluate the Vitamin D level of individuals. According to the Endocrine Society, 25-OH-D =  > 30 ng/ml is considered sufficient; 20–29 ng/ml is evaluated to be inadequate, and < 20 ng/ml is regarded as Deficient Vitamin D status [20, 21]. Therewithal, there are some subgroups for Vitamin D deficiency as Mild, Moderate, and Severe [21]. Identifying serum levels of 25-OH-D needs clinical laboratory resources, so determining this level can be costly to measure. Also, having a Vitamin D test is not always feasible. In addition, in most developing countries, particularly in North Cyprus, Vitamin D testing is not performed in the state laboratories. Thus, this test must be examined in private ones, which may have an additional financial burden for people. Therefore, it may be more convenient to develop an affordable Machine Learning (ML) model to detect Vitamin D status accurately without the need to determine the 25-OH-D level.

Over the last decades, ML as a subset of Artificial Intelligence (AI) has become popular among scholars in various fields, such as engineering, health science, sport science, educational science, etc. [22–26]. ML presents facilities to assist the health sector, particularly physicians, in diagnosing disease more efficiently and with higher precision [27]. Data modeling, and ML techniques can make disease prediction and classification quick and reliable [25, 28]. Supervised classification techniques [29] are modern ML approaches to analyze the response variable in terms of the explanatory variables. According to the literature review, different classification or prediction models using various ML methods have been studied to determine Vitamin D status [30–33]. These comparative studies addressed the binary classification models to classify Vitamin D status [30–33]. However, categorizing the status of Vitamin D as only "Deficient" and "Not Deficient" may cause a loss of information and poor performance due to overgeneralization. Thus, this misleading information may lead to poor estimation. The study [32], discussed ML approaches for predictive models of vitamin D deficiency in a hypertensive population. In this study, Vitamin D level was categorized as binary. The variable selection was performed by the Elastic net method. However, the authors did not clearly emphasize the presence of multicollinearity in the data. Besides, they did not address the effects of the multicollinearity problem on the ML models. Only one study, the [34], attempted to predict (or classify) Vitamin D deficiency by conducting multiclass classification ML methods to classify Vitamin D status. On the other hand, in the study [34], the samples were collected from college students in the age group of 18–21. The authors conducted various classification techniques but did not consider two conventional ordinal logistic and Elastic net ordinal regression models. Often, most data sets used in health science research usually contain highly correlated features, which is referred to as a multicollinearity problem [35]. When two or more features in the model are highly associated with one another, multicollinearity occurs. Namely, multicollinearity presents in the data when two or more features have strong linear relationship. This issue can cause an overfitting problem in the ML training process. In this case, models can perform appropriately on training datasets, despite releasing unacceptable results on unseen testing datasets [36, 37]. Thus, another notable point is an attempt to draw attention to the multicollinearity problem in the self-collected dataset in the current study. In contrast, previous studies did not consider it [30–33].

In the case of the ML model selection, the literature review showed that the Ordinal logistic regression (OLR), Elastic-net ordinal regression (ENOR), Support Vector Machine (SVM), and Random Forest (RF) models are commonly applied to classification problems [30, 32–34, 38–49]. In Table 1, studies using various ML models are summarized according to the metrics used, and the models compared. Moreover, it has been shown that in the literature, the detection and classification of Vitamin D levels based on multicollinearity problems have not yet been considered. Due to the lack of studies in the literature, the proposed study’s purpose is to determine a clinically acceptable ML model for accurately detecting the vitamin D status of the adult participants without the need to determine the 25-OH-D level taking into account the multicollinearity problem. In the current study, the collected dataset consists of the Near East University (NEU) Hospital laboratory test results of the participating applicants in Northern Cyprus.

**Table 1:** Summary of studies using various ML models

Reference no	Authors and Year	Subject	Classification	Method	Metrics	Best model
[32]	Garcia et al. (2021)	Vitamin D	B	LR SVM RF NB XGboost	Accuracy Recall Specificity Predictive values	SVM
[33]	Patino-Alonso et al. (2022)	Vitamin D	B	RF LR NB	Accuracy Error Precision Specificity Recall	LR
[34]	Sambasivam et al. (2020)	Vitamin D	M	KNN DT RF AB BC ET SGD GB SVM MLP	Precision Recall F1-score Accuracy AUC	RF
[42]	Abdullah, Hafidz and Khairunizam (2020)	Chronic kidney disease	B	RF SVMLINEAR SVM NB LR	Accuracy Precision Recall F1-Score	RF
[43]	Xiao et al.(2020)	Alzheimer’s disease	B	LR Proposed LR LR-L_1_ LR-L_2_	Accuracy Recall Specificity	Proposed LR
[44]	Bekele (2022)	Low birth weight	B	LR DT NB K-NN RF SVM Gboost, XGboost	Accuracy Recall Precision F1-Score AUC-ROC	RF
[45]	Kırğıl, et al. (2022)	Diabetes	B	DT NB SVM LR MLP KNN LMT RF	Accuracy Recall	RF
[46]	Ranade (2021)	Inflammatory Bowel Disease from vitamin D	M	DT SVM ET	Accuracy AUC	DT
[47]	Wainer et al. 2016	NA	B	BST ELM GBM ENLR KNN LVQ NB NNET RF RKNN KNN SDA SVMLINEAR SVMPOLY SVM	Error Rate Bayesian ANOVA Training and Testing time	RF GBM SVM
[48]	Deist et al. (2018)	Radiation treatment	B	DT RF ANN SVM ENLR Logit-Boost	Calibration Accuracy Cohen’s kappa AUC Brier score	RF ENLR
[49]	Abdullah et al. (2022)	Alzheimer’s disease	B	Lasso LR Ridge LR ENLR NB SVM K-NN RF	Recall Precision Accuracy F1-Measure	ENLR RF

*M* Multiclass, *B* Binary, *ENLR* Elastic-net logistic regression, *BST* Boosting of linear classifiers, *ELM* Extreme learning machines, *ENLR* Elastic net logistic regression, *GBM* Gradient boosting machines, *KNN* k-nearest neighbors classifier, *LMT* Logistic Model Tree, *LVQ* Learning vector quantization, *LR* Logistic Regression, *MLP* Multilayer Perceptron, *NB* Naive Bayes classifier, *NNET* 1-hidden layer neural network with sigmoid transfer function, *RF* Random forest, *RKNN* A bagging of KNN classifiers on a random subset of the original features, *SDA* L1 regularized linear discriminant classifier, *SVM* SVM with RBF kernel, *SVMLINEAR*: SVM with linear kernel, *SVMPOLY* SVM with polynomial kernel, *Xgboost* Extreme Gradient Boost

### Motivation

**Fig. 1:**
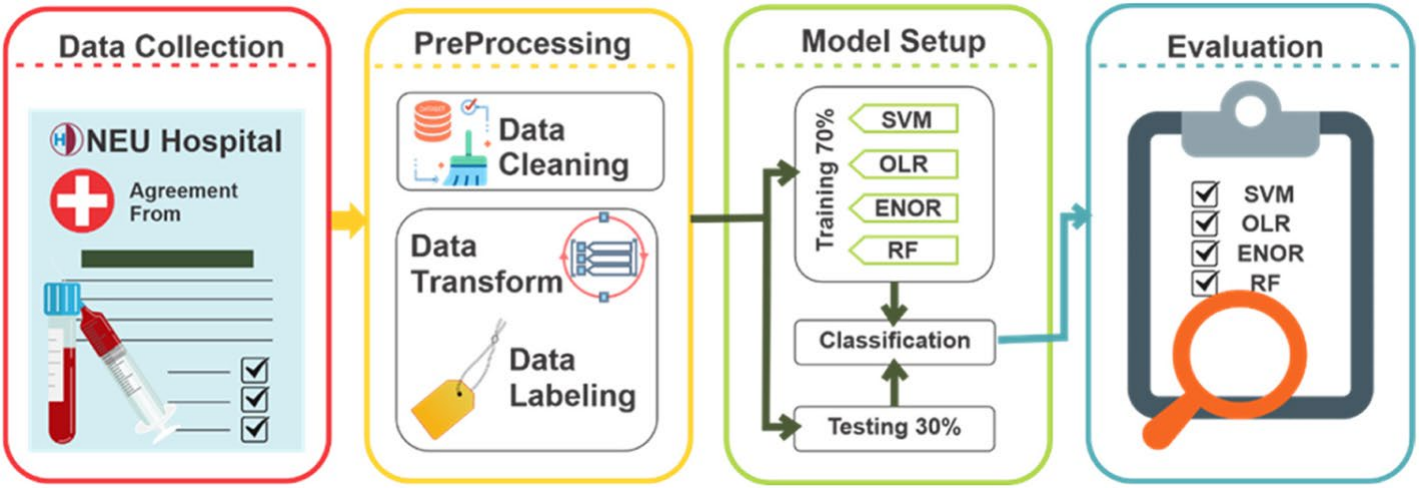
The block diagram of the study

In the big picture, the author's perspective is developing a Vitamin D-detecting application with a user-friendly graphical user interface as the first phase of the computer-aided system for diagnosing diseases caused by Vitamin D deficiency. The three main classes, namely, "Deficiency," "Adequate," and "Inadequate," are considered as the level of Vitamin D that are detected and classified in the current study [20]. Figure 1 depicts the block diagram of the study. The system can provide reports based on the level of Vitamin D for treatment staff, particularly physicians.

### Contributions

The primary contributions of this study are as follows: (a) A detailed comparison of the model's performances based on the four examinations is provided: 1) Observe sensitivities of the selected models to the participants' MtS status 2) Observe the parameter tuning procedures of the models 3) Observe the sensitivities of the selected models to the dataset where the models are trained, and 4) Compare the classification performances of the selected models. The current study's criteria selection phase was released earlier in the recent publication based on the same ethics committee code [43]. A multiclass classification model that receives the laboratory test results as inputs and classifies the level of Vitamin D in three groups at the output, is determined. (c) The effect of the multicollinearity problem on the self-collected data, as health data, on the proposed models' behaviors and performance was examined and proved.

## Methods

The data collection protocols, participants' characteristics, computational analysis, and the basics of the ML algorithms are briefly described in this section.

### Data collection protocols

The members of two groups have collected the dataset: 1) the volunteer applicants and 2) the physicians. The data collection phase was started after the NEU scientific ethics committee approval and was terminated at the end of July 2022. The volunteer applicants delivered their signed agreement forms in advance during the data collection process. Coincidently, in this study, there are more female applicants than males. Thus, gender factor equality is not provided. In the case of the physicians, two internists whom NEU Hospital Internal Medicine Department introduced acted as supervisors. They supervised data collection and labeled each took laboratory result of Vitamin D level individually. Besides, the physicians also contributed to the determination of the presence of the MtS on the self-collected dataset during the data collection phase. During this time, 512 observations were collected from people over 18 years old who applied voluntarily to Internal Medicine Department at NEU Hospital. Ethical approval for this research was obtained from NEU Scientific Research Ethics Committee (Date: April 29, 2021, Decision No: YDU/2021/90–1327) [50]. By comparing the number of samples with the latest publication [50] based on the same Ethics Committee, 156 observations were added to the dataset.

### ML models

According to the self-collected data origin, the dataset includes highly correlated features to each other, which is addressed the multicollinearity problem. Literature review showed that in the case of multiclass classification, OLR, ENOR, RF, and SVM are applied. The current study examines the multicollinearity problem effects on the models' performances.

### Ordinal Logistic Regression (OLR)

OLR is a generalized linear model that is applied to predict an ordinal response variable given one or more predictors. Using classical regression models for the cases where the dependent variable is ordinal categorical may cause a non-linear relationship between the independent variables and misinterpretation of the estimated probabilities. The OLR is widely used in studies when the dependent variable is ordinal categorical. This model provides the assumption of parallelism between the categories. This cumulative logit model is also called the proportional odds model, defined in Eq. (1) [51, 52].

1 $$log\left[\frac{P\left(y\le {y}_{j}|x\right)}{P\left(y>{y}_{j}|x\right)}\right]={\alpha}_{j}-({\beta}_{0}+{\beta }_{1}{x}_{1}+{\beta }_{2}{x}_{2}+\dots +{\beta }_{p}{x}_{p}),\mathrm{ j}=\mathrm{1,2},\dots,\mathrm{J}-1$$

The model is based on the distribution of cumulative probabilities. In the model, ${\pi}_{j}=P\left(y\le {y}_{j}|x\right)$ is the cumulative probability of the event,$y\le {y}_{j}$. ${\beta }_{1},{\beta }_{2},\dots .,{\beta }_{p}$ are the unknown regression coefficients, ${x}_{1},{x}_{2},\dots .,{x}_{p}$ are explanatory variables, and p is the number of explanatory variables. The OLR will have J-1 intercepts, denoted by ${\alpha }_{j}$ such that ${\alpha }_{1}\le {\alpha }_{2}\le \dots .\le {\alpha }_{J-1}$. This allows for the intercept to differ for each cumulative logit. For the proportional odds model, each cumulative log has its own cutoff point. For all cumulative logits, the βs of the independent variables are equal. Thus, it provides the assumption of parallelism and the presence of multicollinearity that must be tested. Brant’s Wald Chi-square test is used to test the assumption of parallelism in the ordinal regression models [52].

### Elastic-net Ordinal Regression (ENOR)

ENOR is a penalized regression model that linearly connects L1 and L2 penalties. The model is a combination of the strengths points of the Lasso and Ridge regression methods [53]. The Elastic net method has two tuning hyper-parameters, namely, a mixing parameter (α) and a regularization parameter (λ) to balance between the Lasso and the Ridge regression methods [54]. The regularization term in the ENOR model is a simple mixture of both Ridge's and Lasso's regularization terms, and the mixing ratio can be controlled by the coefficient alpha (α). When the α = 0, the ENOR model is equivalent to the Ridge Regression. When α = 1, ENOR is equivalent to the Lasso Regression. The penalized objective function for ENOR in parallel form is exposed in Eq. 2 [55]:

2 $${argmin}_{\beta }\left\{-\frac{1}{{N}^{*}}L\left({\beta }_{0},\beta \right)+\lambda {\sum }_{i=1}^{p}(\alpha \left|{\beta }_{i}\right|+\frac{1}{2}(1-\alpha ){\beta }_{i}^{2}\right\} i=\mathrm{1,2},\dots ,p$$

$$L\left(.\right),$$$$\alpha and$$$$\lambda$$ are the model setting parameters where $$L\left(.\right)$$ is the log-likelihood, the $$\lambda$$ value has to be greater thanor equal to zero ( $$\lambda \ge 0$$) and the $$\alpha$$ is between or equal to 0 and 1 $$(0\le \alpha \le 1)$$.$${N}^{*}$$ is the sum of the ordinal trials and $${\beta }_{i}$$ is the ith element of the vector of coefficients $$\beta$$. ENOR as a supervised model is one of the regularized ML models and is not affected by the multicollinearity problem. It gives precise results in the presence of this problem in the dataset.

### Random Forest (RF)

RF method was proposed by Brieman in 2001 [56] as a supervised classification algorithm. The RF is a powerful nonparametric statistical method; this model takes advantage of the combination of the Decision Tree, Bagging, and Random Subspace methods. RF is frequently used in regression problems and for binary and multiclass classification problems as well. According to his origin as a community formed by many decision trees, it is called a Random Forest. Each dataset is generated by displacement from the original dataset. Trees are then developed using the Random Feature selection method. The developed trees are not pruned. This strategy makes a unique accuracy for the RF [57]. The RF is also very fast, resistant to overfitting, and can be applied with as many trees as desired [56]. To identify an appropriate RF model, two setting hyper-parameters must be tuned in advance. The number of features ($q$) which is referred to use at` each node to determine the determining best split for each node, and the ($N$) which is depicted as the number of trees.

In advance, the bootstrapping samples are allocated from 2/3 of the dataset samples as the training dataset. The remaining 1/3 of the dataset, also called out-of-bag (OOB) data, is used to test for errors. The tree is then developed without pruning from each preloaded sample. At each node, $m$ variables are randomly selected among all variables and the best branch is defined among them. This algorithm has a direct relationship between the number of trees and the result. In other words, by increasing the number of trees, we can get closer to the precise results. On the other hand, the overfitting problem is a critical issue that adversely affects the results. However, the RF algorithm reduces the probability of an overfitting problem if there are enough trees in the forest.

### Support Vector Machine (SVM)

SVM as a supervised ML algorithm can solve regression and classification tasks in binary and multiclass types. This model can separate data by using hyperplanes [58]. The main feature of the optimum hyperplane is best fits the data. Similar to the other classification methods, the outliers, if anyone exists, may affect the optimum hyperplane. In this regard, to add a non-linearity possibility, one of the kernel function types (i.e., Linear, Radial, Sigmoid, and Polynomial) is integrated into the model. In other words, the SVM model can classify data into two types: linear and non-linear. In the case of linear classification, the binary outcome is the assumption of the problem. The model plots data points in space and separates the values by an explicit gap. Thus, the model predicts the optimum hyperplane to divide data into two classes [59]. The main attribute of the optimum hyperplane is the maximum space between the plane and the closest data point of either class. This attribute is the main reason for naming the plane as a maximum-margin hyperplane. When data cannot be separated in a finite space, a conventional linear hyperplane might not be an appropriate plane to classify. Thus, a non-linear type is acted and classifies the data by using one of the kernel function types.

In Table 2, distinctive features of the ML models used in the study have been discussed in detail. OLR, ENOR, and RF are interpretable. However, SVM lacks interpretability because of the complexity of the learned hyperplane [60]. Furthermore, all the models used in the study have the robustness feature [61, 62]. On the other hand, when we examine the scalability feature for the models, we observe that SVM and RF are scalable for high-dimensional datasets whereas OLR is not scalable for high-dimensional datasets because of the complexity in optimization [63]. ENOR is more scalable than OLR due to regularization [64]. Moreover, due to the model structure, multicollinearity significantly affects OLR. High multicollinearity results in inflated standard errors of the coefficients, erroneous findings, and overfitting in OLR. This might result in inaccurate generalization to new data. Also, overfitting in SVM is more likely in classification. Especially, a small sample size, may make an SVM more likely to overfit in classification, which might produce false diagnostic findings [65, 66]. A regularization term that indicates the complexity of the model is included in the objective function of the SVM in addition to a loss function that measures the correctness of the fitting. Therefore, the optimization objective is to avoid creating complicated models that may result in overfitting because of the pursuit of local optima by taking into account both the structural risk of the model as well as the empirical risk [67]. However, ENOR is one of the regularized ML models and is not affected by the overfitting and multicollinearity problem because of the combination of L1 and L2 regularizations. It gives precise results in the presence of these problems in the dataset. RF is also proposed to reduce overfitting by ensemble learning [56, 67, 68]. Alternatively, all models except OLR have hyperparameters that need tuning.

**Table 2:** Distinctive features of OLR, ENOR, RF, and SVM

Distinctive Features	OLR	ENOR	SVM	RF
Interpretability	✔	✔	X	✔
Hyper-parameter tuning	X	✔	✔	✔
Controlling overfitting	X	✔	X	✔
Flexibility ( dealing with datasets that have many correlated predictors)	X	✔	X	✔
Multiclass capability	✔	✔	✔	✔
Robustness to noise and outliers	✔	✔	✔	✔
Efficiency (scalability for high-dimensional dataset)	X	✔	✔	✔

✔: Exists X: Does not exist

### Performance measurements of the ML models

**Fig. 2:**
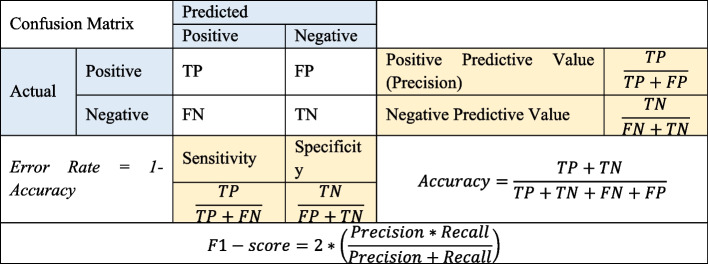
Performance evaluation metrics using confusion matrix

Various criteria evaluated the classification performances of the ML models on the testing dataset in order to determine the most appropriate model when the model's training is completed. Mainly, the confusion matrix is applied to evaluate the models' classification performance which compares the actual and predicted values. The confusion matrix is a table with rows and columns that describes the frequency of *True Positives (TP)*, *False Negatives (FN)*, *False Positives (FP)*, and *True Negatives (TN*) as seen in Fig. 2. In the case of imbalanced datasets, as is the case with the collected data in this study, model selection based on the accuracy metric individually, can lead to misleading results. Therefore, the F1-score is a valuable metric to measure the model’s performance. Other performance metrics such as Specificity, Sensitivity, Precision, Accuracy, Error Rate, and Cohen’s kappa ($k$) which are calculable from the matrix, might present a fair comparison vision for the authors in the current study.

Accuracy value is an overall measure of the ratio of correctly predicted observations in the model to the total number of observations. The bigger number in the accuracy metric results may depict the more successful model. Precision as TP ratio corresponds to the proportion of positive data points considered positive relative to all positive data points. Besides, Recall (or Sensitivity) metric indicates how well Positive states are predicted. Specificity as FP rate corresponds to the proportion of negative data points considered FP with respect to all negative data points. Finally, F1-score is a harmonic mean of Precision and Sensitivity metrics that can measure the models’ accuracy. Cohen`s kappa (inter-observer agreement) is used to analyze the inter-class accuracies obtained from the confusion matrix for multiclass classification problem. This coefficient takes values between 0 and 1 ($0\le k\le 1$) and is calculated using the row, and column totals of the confusion matrix and the elements on the diagonal. The released result by the kappa is interpreted as follows: when the $k$ value is between 0.81 and 1 indicates almost perfect agreement; between 0.61 and 0.8 as substantial agreement; in the range of 0.41 and 0.6 as moderate agreement; between 0.21 and 0.4 as fair agreement, in the range of the 0.01 and 0.20 as none to a slight agreement, and when ($0\le k\le 0.01)$ as exposing no agreement.

### Computational analysis

In the case of the OLR and ENOR models implementation, R Studio version 2021.9.0.351 (RStudio 2015) with the ordinal, ordinalNet, MASS, ordinalgmifs, brant, mctest, dplyr, tidyverse, caret, boot packages, has been conducted for computational analysis. In the case of the RF, and SVM implementation, we applied the Python library. A computer equipped with 3.7 GHz i7-8700 k core processors, 32 G RAM, and NVIDIA 1080 Ti GPU trained all models offline.

### Experimental setup

Due to the exclusion criteria exposed and approved by the physicians, some of the observations were excluded. The list of exclusion criteria is explained in detail in the authors' previous article [50]. In the case of the features selection, twenty-two features were included and were registered namely, Waist circumference (WC), body mass index (BMI), uric acid level (UAL), Systolic blood pressure (SBP) and Diastolic blood pressure (DBP), lipid panel measurements (Low-density lipoprotein (LDL)-cholesterol, triglycerides (TRY), High-density lipoprotein (HDL)-cholesterol, Total Cholesterol Level (TCL), High-sensitivity C-reactive protein (hs-CRP), homeostatic model assessment–insulin resistance (HOMA-IR), fasting blood sugar (FBS) and 25-OH Vitamin D levels of the individuals. Moreover, the information of the participants about their gender, age, having smoke and alcohol, MtS, skin tone, usage of sun protection cream, and sunlight exposure status, use of daily multivitamin and mineral supplement (MMS), and usage of fish oil supplement (FOS) were collected. During the data collection phase, the eating habits factor such as consumption of salmon, consumption of egg folk, and consumption of milk and its products had not been appropriately collected. Thus, cause of the misleading observations and incomplete information, the eating habit factor has not been included. Table 3 shows the clinical and lifestyle characteristics of the training and testing dataset participants.

**Table 3:** The clinical and lifestyle characteristics of the participants of the training and testing datasets

Features		Training data(*n* = 337)	Testing data(*n* = 144)	Whole data(*n* = 481)
		**N (%)**	**N (%)**	**N (%)**
**Gender**	**Female**	210(62.3)	110(76.4)	320(66.5)
	**Male**	127(37.7)	34(23.6)	161(33.5)
**Vitamin D status**	**Deficient**	192(57.0)	82(57.0)	274(57.0)
	**Inadequate**	81(24.0)	34(23.6)	115(23.9)
	**Adequate**	64(19.0)	28(19.4)	92(19.1)
**Age groups**	**18–29**	10(3.0)	5 (3.5)	15(3.1)
	**30–39**	56(16.6)	21(14.6)	77(16.0)
	**40–49**	92(27.3)	43(29.9)	135(28.0)
	**50–59**	117(34.7)	54(37.5)	171(35.6)
	**60–69**	37(11.0)	14(9.7)	51(10.6)
	**70 + **	25(7.4)	7(4.9)	32(6.7)
**SBP**	**Normal level**	164(48.7)	63(43.8)	227(47.2)
	**High level**	173(51.3)	81(56.3)	254(52.8)
**DBP**	**Normal level**	219(65.0)	95(66.0)	314(65.3)
	**High level**	118(35.0)	49(34.0)	167(34.7)
**FBS**	**Normal level**	201(59.6)	82(56.9)	283(59.8)
	**High level**	136(40.4)	62(43.1)	198(41.2)
**HOMA-IR**	**Normal level**	196(58.2)	70(48.6)	266(55.3)
	**High level**	141(41.8)	74(51.4)	215(44.7)
**hs-CRP**	**Normal level**	210(62.3)	65(45.1)	275(57.2)
	**High level**	127(37.7)	79(54.9)	206(42.8)
**UAL**	**Normal level**	231(68.5)	81(56.3)	312(64.9)
	**High level**	106(31.5)	63(43.7)	169(35.1)
**HDL**	**Low level**	179(53.1)	78(43.8)	257(53.4)
	**Normal level**	158(46.9)	66(56.3)	224(46.6)
**TCL**	**Normal level**	195(57.9)	71(49.3)	266(55.3)
	**High level**	142(42.1)	73(50.7)	215(44.7)
**LDL**	**Normal level**	173(51.3)	79(54.9)	252(52.4)
	**High level**	164(48.7)	65(45.1)	229(47.6)
**TRY**	**Normal level**	201(59.6)	92(63.9)	293(60.9)
	**High level**	136(40.4)	52(36.1)	188(39.1)
**MtS**	**No**	186(55.2)	64(44.4)	250(52.0)
	**Yes**	151(44.8)	80(55.6)	231(48.0)
**Having alcohol**	**No**	244(72.4)	101(70.1)	345(71.7)
	**Yes**	62(18.4)	24(16.7)	86(17.9)
	**Used before**	31(9.2)	19(13.2)	50(10.4)
**Having smoke**	**No**	261(77.4)	93(64.6)	354(73.6)
	**Yes**	76(22.6)	51(35.4)	127(26.4)
**Skin tone**	**Light**	156(46.3)	62(43.1)	218(45.3)
	**Dark**	181(53.7)	82(56.9)	263(54.7)
**Usage of sun protection cream**	**No**	193(57.3)	89(61.8)	282(58.6)
	**Yes**	144(42.7)	55(38.2)	199(41.4)
**Sunlight exposure status**	**No direct exposure to the sun**	275(81.6)	94(65.3)	369(76.7)
	**Direct exposure to the sun**	62(18.4)	50(34.7)	112(23.3)
**Usage of MMS**	**No**	271(64.4)	112(77.8)	383(79.6)
	**Yes**	66 (19.6)	32(22.2)	98(20.4)
**Usage of FOS**	**No**	304(90.2)	120(83.3)	424(88.1)
	**Yes**	33(9.8)	24(16.7)	57(11.9)
**WC**	**Normal to moderate central fat accumulation**	156(46.3)	53(36.8)	209(43.5)
	**High central fat accumulation**	181(53.7)	91(63.2)	272(56.5)
**BMI**	**Not having obesity**	185(54.9)	77(53.5)	262(54.5)
	**Having obesity**	152(45.1)	67(46.5)	219(45.5)

### Data preprocessing

The original raw self-collected dataset consisted of 512 samples. As observed in Fig. 1, the pre-processing phase includes two main stages: 1) Data cleaning and 2) Data transformation. In the case of the first stage, the participants' laboratory test which did not include the level of the Vitamin D results, were eliminated from the dataset. Thus, 31 samples were excluded. A final dataset of 481 laboratory test results was used as the self-collected dataset. Converting the continuous test result to categorical data by labeling them is called data transformation. In this stage, the physicians label the continuous results of laboratory tests and convert them to categorical data based on the laboratory test indicators' reference values. As a result, the dataset with the continuous variable is converted to the categorical ones. According to Table 3, 57.0% are allocated to the "Deficiency" class, 19.1% of them are "Adequate," and the rest (23.9%) is allocated to the "Inadequate" class. Thus, the self-collected dataset's statistical characteristics showed that the dataset is not balanced. All variables' threshold values are described as follows:

According to the 25-OH Vitamin D (VD) levels, participants were separated into three classes as the Deficient Vitamin D class (VD < 20 ng / mL), Inadequate Vitamin D class (20 ng/mL <  = VD < 30 ng / mL), and Adequate Vitamin D class (VD >  = 30 ng/mL). Participants with TCL greater than 200 mg / dL were evaluated as having “High Cholesterol.” A level of HOMA-IR greater than 2.5 was evaluated as “Insulin Resistance”. The level of LDL greater than 130 mg / dL was evaluated as “High LDL.” In addition, participants with UAL greater than 6 mg / dL for females and UAL greater than 7 mg / dL for males were taken as “High Level for Uric Acid”, and the levels of hs-CRP greater than 0.5 mg / dL were evaluated as “High hs-CRP”. Moreover, MtS was defined according to the National Cholesterol Education Program (NCEP) Adult Treatment Panel III (ATP III) identification [69]. Based on this description, MtS is present if three or more of the following five criteria are provided: WC over 102 cm (male) or 88 cm (female) as high central fat accumulation, blood pressure (SBP/DBP) over 130/85 mmHg, fasting TRY level over 150 mg/dl, fasting HDL cholesterol level less than 40 mg/dl (men) or 50 mg/dl (women) and fasting blood sugar over 100 mg/dl. Besides, having obesity degree of the participants was evaluated with BMI value. BMI is greater than 30 $\frac{\mathrm{kg}}{{\mathrm{cm}}^{2}}$ for the participants who are obesity.

### Predictors selection

In the current study, based on the categorical type of all variables, the predictors’ selection has been performed by Pearson’s chi-squared (${\upchi }^{2})$ test as the Filter method. The results of the test can identify the dependencies between the categorical variables. The p-value results are a decision tool to determine whether the categorical predictors are correlated with outcome variables, the level of Vitamin D, or not. When the *P*-value < 0.010, it seems certain that there is a correlation between the level of Vitamin D status and the categorical predictors. As seen in Table 4, according to the ${\upchi }^{2}$ test results on the whole samples of the dataset (*n* = 481), we can claim that there is a statistically significant correlation between the level of Vitamin D status and the below-mentioned predictors since *P* < 0.01: FOS (P < 0.001), MMS ((*P* < 0.001), sunlight exposure status(*P* < 0.001), skin tone(*P* < 0.001), HDL (*P* < 0.001), hs-CRP (*P* < 0.001), WC (*P* < 0.001), BMI (*P* < 0.001), TRY (*P* < 0.001), UAL (*P* = 0.002), MtS (*P* < 0.001), age groups (*P* = 0.002). Their statistically significant Gamma (G) correlation coefficient is shown in Table 5 as the highest negative correlation among the calculated correlations was between the presence of MtS and Vitamin D class (G = -0.719, *P* < 0.001).

**Table 4:** Pearson Chi-square(${\chi }^{2}$) test for independence between various predictors and Vitamin D classes

**Predictors**	${\chi }^{2}$(df)^a^	*P* value	**Predictors**	${\chi }^{2}$(df)^a^	*P* value
Gender	1.622(2)	0.444	TRY	57.296 (2)	< 0.001
Age groups	27.743 (10)	0.002	MtS	70.231 (2)	< 0.001
SBP	2.630 (2)	0.268	Having alcohol	5.501(2)	0.064
DBP	3.485 (2)	0.175	Having smoke	3.796 (2)	0.150
FBS	2.516 (2)	0.284	Skin Tone	60.897 (2)	< 0.001
HOMA-IR	0.666 (2)	0.717	Usage of sun protection cream	4.124 (2)	0.127
hs-CRP	28.509 (2)	< 0.001	Sunlight exposure status	14.094(2)	< 0.001
UAL	12.405 (2)	0.002	Usage of MMS	31.727(2)	< 0.001
HDL	42.633 (2)	< 0.001	Usage of FOS	26.390 (2)	< 0.001
TCL	2.907(2)	0.234	WC	45.587 (2)	< 0.001
LDL	1.786 (2)	0.409	BMI	57.004(2)	< 0.001

^a^*df* Degrees of freedom

**Table 5:** Gamma correlation coefficient $(G)$ between various predictors and Vitamin D classes

**Predictors**	$G$	*P* value	**Predictors**	$G$	*P* value
Gender	0.102	0.563	TRY	-0.520	< 0.001
Age groups	0.203	< 0.001	MtS	-0.719	< 0.001
SBP	-0.130	0.105	Having alcohol	-0.135	0.070
DBP	-0.150	0.088	Having smoke	-0.102	0.201
FBS	-0.049	0.560	Skin Tone	-0.414	< 0.001
HOMA-IR	0.027	0.735	Usage of sun protection cream	-0.121	0.203
hs-CRP	-0.488	< 0.001	Sunlight exposure status	0.372	0.007
UAL	-0.438	< 0.001	Usage of MMS	0.210	< 0.001
HDL	0.453	< 0.001	Usage of FOS	0.247	< 0.001
TCL	0.132	0.101	WC	-0.487	< 0.001
LDL	0.068	0.375	BMI	-0.602	< 0.001

### Parameter tuning and model setups

It has been asserted that [24], the models with fine-tuning could achieve better results. In this regard, setting parameters before training could achieve the desired results in the case of the study’s proposed RF, ENOR, and SVM models. In the case of the ENOR, *K*-fold cross-validation (*K* = 5) was implemented to set the model parameters. However, in the case of the other two models, RF and SVM, there is no commonly accepted technique for choosing the appropriate hyper-parameters’ values. Applying a trial and error strategy, the validation dataset, and using the results of similar studies are the most universally accepted methods [70]. In the case of the RF, the number of features ($q$) is selected from the interval [1, $\sqrt{p}$] where $p$ is the number of predictors in the model. Table 6 depicts the proposed ENOR, RF, and SVM models range of the hyperparameters and their optimum value.

**Table 6:** The proposed ENOR, RF, and SVM models range of the hyper parameters and the optimum value of them

Models	Hyper-parameters	Value range	Optimum values
OLR	-	-	-
ENOR	$\alpha$	[0, 1]	0.1000
	$\lambda$	${lambdaminratio=\lambda }_{min}/{ \lambda }_{max}$ lambdaminratio = 0.01	0.0325
SVM	$C$	10^0^, 10^1^, 10^2^, …,10^5^	10^2^
	$Gamma$	10^0^, 10^–1^, 10^–2^, …, 10^–7^	10^–7^
	$kernel$	Linear, Radial Basis Function (RBF), Polynomial	RBF
RF	$q$	[1, 4]	4
	$N$	[10,500]	300

Overfitting problems emerge through the training phase. To benchmark the proposed models' performance fairly and avoid the overfitting problem, the self-collected dataset was selected randomly and divided into 30% as a testing set, and the rest of the data was allocated as a training dataset. The proposed models' performances are evaluated based on the performance measurement metrics. The training set is in charge of developing models and testing ones is in charge of evaluating the models' performance. The validation dataset, which is 5% of the training set, is used for parameter tuning. According to the study [34], Vitamin D level was divided into four main categories: Sufficiency, Insufficiency, Deficiency, and Severe Deficiency. However, in our data, based on the inadequate number of the samples in "Deficiency" and "Severe Deficiency" subgroups, the physicians classified these samples into the "Deficiency" level. On the other hand, the inadequate number of samples in each category may affect the determined ML models' average classification results. Thus, in this study, according to Endocrine society reports [20], and the contributed physicians' point of view, the level of Vitamin D is categorized into three main classes, namely: Adequate, Inadequate, and Deficiency.

The current study applies the Random Selection method to select the training and testing samples. On the other hand, the self-collected dataset is not balanced. This means that the sample distributions are not equal in the three classes. As seen in Table 3, the Deficiency class with 274 samples (57% of the dataset) is the greater one which may lead to a positive class problem when applying classification [32]. Thus, a different validation technique is necessary. In order to overcome this challenge and prevent any misleading effect on the results, the authors allocated a balanced ratio of all classes when the samples were randomly selected for the training and testing datasets. Accordingly, the proportions of the Vitamin D classes' samples are balanced in the datasets.

### Detection of the multicollinearity problem

As a first step of the model choosing and setting, checking the multicollinearity problem existence is inevitable. Multicollinearity problem presents in the dataset if there are high linear inter-correlation between explanatory variables and causes models to give misleading results. The presence of multicollinearity in the data is generally determined by diagnostic measures, namely: Variance Inflation Factor (VIF), Condition Index (CI), and Variance Decomposition Proportions (VDP). When the VIF is greater than 4 to 10 and/or CI is greater than 30, it is concluded that there is multicollinearity between the variables [71, 72]. Besides, the VDP is evaluated to determine variables that are multicollinear. If VDP is greater than 0.8 to 0.9 and CI is greater than 30, the explanatory variables providing this condition are determined to be multicollinear [73]. According to Table 7, the multicollinearity problem presents in the data. As a result, VIF values of the variables TRY, MtS, BMI, and WC are greater than 4, which are 5.589, 12.567, 25.214, and 28.396, respectively, as seen in Table 5. Moreover, three CI values (CI_11_ = 32.855, CI_12_ = 59.620, CI_13_ = 103.443) greater than 30 show that there is a severe multicollinearity problem. In order to determine which variables are multicollinear in the data, VDP values were examined as well. As seen in Table 7, in the case of VDP, when the VDP values corresponding to the CI greater than 30 are observed, it is concluded that there is a strong relationship between the variables TRY, MtS, BMI, WC, since the VDP values of these variables are greater than 0.8.

**Table 7:** Collinearity diagnostics

Eigenvalue	Condition Index	Variance Decomposition Proportions											
		**MMS**	**Sunlight Exposure Status**	**FOS**	**TRY**	**HDL**	**hs-CRP**	**BMI**	**WC**	**SKIN TONE**	**AGE**	**MtS**	**UAL**
4.645	1.000	0.004	0.001	0.003	0.001	0.003	0.004	0.002	0.001	0.003	0.003	0.002	0.001
1.818	2.555	0.028	0.007	0.001	0.003	0.004	0.017	0.001	0.003	0.010	0.118	0.028	0.005
1.387	3.349	0.029	0.002	0.000	0.000	0.014	0.149	0.001	0.006	0.153	0.205	0.028	0.001
1.141	4.072	0.011	0.004	0.066	0.003	0.058	0.043	0.008	0.009	0.063	0.381	0.003	0.006
0.756	6.144	0.166	0.001	0.015	0.002	0.007	0.002	0.013	0.001	0.062	0.043	0.000	0.001
0.513	9.051	0.393	0.010	0.077	0.007	0.000	0.002	0.010	0.005	0.002	0.046	0.004	0.007
0.434	10.693	0.352	0.005	0.414	0.001	0.000	0.187	0.009	0.004	0.063	0.135	0.007	0.002
0.393	11.835	0.002	0.003	0.007	0.000	0.005	0.004	0.000	0.002	0.253	0.037	0.001	0.006
0.362	12.822	0.009	0.212	0.294	0.001	0.092	0.008	0.019	0.034	0.256	0.008	0.014	0.011
0.286	16.264	0.002	0.348	0.006	0.005	0.019	0.000	0.018	0.001	0.004	0.004	0.013	0.558
0.141	32.855	0.004	0.171	0.002	0.056	0.682	0.579	0.002	0.006	0.000	0.018	0.001	0.100
0.078	59.620	0.000	0.010	0.079	**0.819**	0.107	0.000	0.000	0.004	0.113	0.000	**0.876**	0.040
0.045	103.443	0.000	0.227	0.036	0.102	0.009	0.006	**0.916**	**0.923**	0.016	0.002	0.022	0.263
**VIF**		1.256	1.859	1.548	**5.589**	2.105	1.282	**25.214**	**28.396**	1.254	1.763	**12.567**	2.141

## Results

### Remarkable observations

The performances of four conventional supervised classification models, namely OLR, ENOR, SVM, and RF, in determining the Vitamin D level, are compared. Based on the theoretical analysis and experimental results, four essential observations that can be obtained are illustrated below:

### O1. Sensitivity to the MtS’ status

**Fig. 3:**
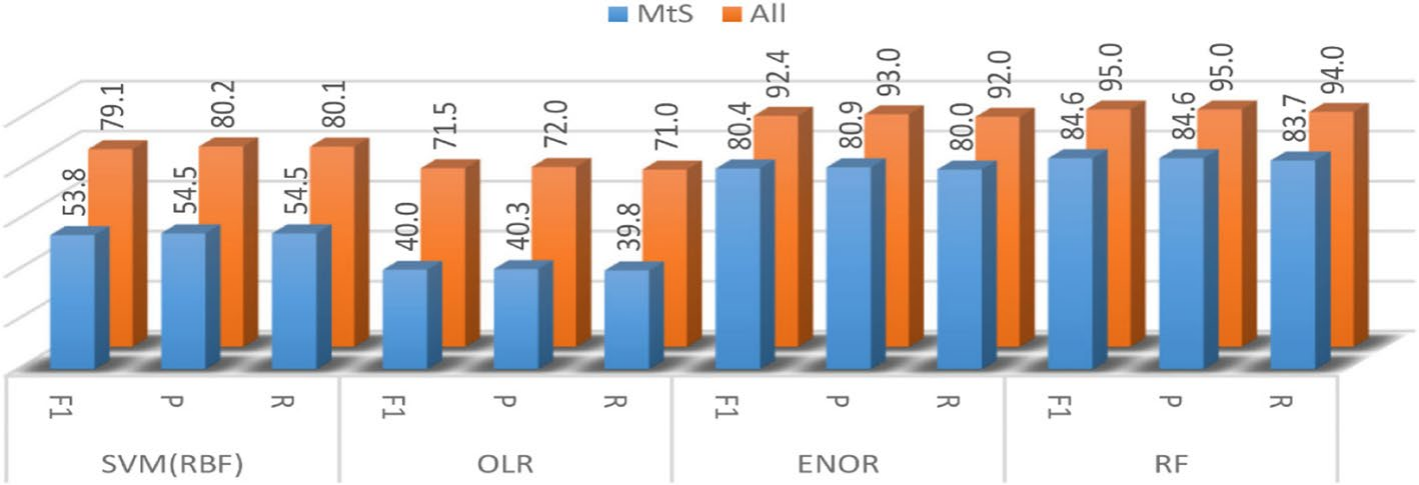
The sensitivities to the MtS’ status

The sensitivities of the developed models to the results of the MtS' status were examined to assign which of the models is more robust to the results of this factor. The literature review revealed a significant relationship between the existence of MtS and Vitamin D deficiency [74, 75]. Besides, when the correlation coefficient values between the Vitamin D classes and the variables were examined, it was determined that the highest negative correlation among the calculated correlations was between the presence of MtS and Vitamin D class on the dataset (G = -0.719, *p* < 0.001). According to National Cholesterol Education Program (NCEP) definition [69], five main criteria have affected the status of the MtS: fasting triglyceride (TG) level over 150 mg/dl, waist circumference over 88 cm (women) or 102 cm (men), fasting high-density lipoprotein (HDL) cholesterol level less than 50 mg/dl (women) or 40 mg/dl (men), fasting blood sugar over 100 mg/dl and blood pressure over 130/85 mmHg. In this regard, if three or more of the five criteria mentioned above are met, the status of the MtS is Positive (1), and if not is Negative (0). During the data collection phase, MtS status, as one of the main features of the study, is considered and classified by the physicians who participated in the current study based on the laboratory test results. Figure 3 shows how the classification performances of all models are remarkably affected by the MtS' positive status (1) on the testing dataset samples. For the sensitivity of the model to the MtS' Positive status, the models have only trained with 100% of the MtS' Negative status (0) applicants group samples and with randomly selected samples containing 20% of those in MtS' Positive status. The models were tested on the rest part of the Mts' Positive status group samples. The weighted mean of the F1-score for the proposed models' performance is 79.1% for the SVM(RBF), 71.5% for the OLR, and 92.4% and 95.0% for the ENOR and RF models, respectively when examining the model's performances on over all samples with random selection strategy to select the training and testing datasets. In the case of SVM (RBF), the weighted mean of the F1-score decreased from 79.1% to 53.8%, and for the OLR model dropped from 71.5% to 40.0%. The models' weighted mean of the F1-score decreased from 92.4% to 80.4% and from 95.0% to 84.6% for the ENOR and RF, respectively. The models' weighted mean of Recall (R) decreased from 80.1% to 54.5% for the SVM(RBF) and dropped from 71.0% to 39.8% for the OLR. In the case of ENOR and RF, the weighted mean of Recall decreased from 92.0% to 80.0% and decreased from 94.0% to 83.7%, respectively. The weighted mean of precision(P) for models decreased from 80.2% to 54.5% for the SVM(RBF), from 72.0% to 40.3% for the OLR, from 93.0% to 80.9% for the ENOR, and from 95.0% to 84.6% for the RF. As seen in Table 7, the MtS predictor is a multicollinear predictor. The proposed RF and ENOR are approximately 31% more robust to the MtS' Positive status than the OLR model.

Due to the presence of multicollinearity, the observations showed that the performance of the optimum SVM(RBF) is significantly negatively affected when the test is examined in the case of the SVM(RBF). However, the performances of ENOR and the RF are not significantly affected when the MtS' status is positive. The performance of ENOR dropped by 13%, and the performance of RF reduced by 11% when the test was examined. In other words, these two models are not affected by the presence of the multicollinearity problem.

### O2. Parameter tuning

According to the parameter tuning section, all three models, namely RF, ENOR, and SVM(RBF), need to tune at least two hyper-parameters to obtain the optimum models. In most cases, the best parameters are tuned based on the other research results in similar cases by implementing the validation dataset and/or trial and error strategies. In this case, all methods may need to use lots of time and cost to achieve optimum values for the hyper parameters. However, in the case of the OLR, this model does not have any hyper parameter to tune. Thus, applying the OLR is less challenging than the others. The models' weighted means of F1-score values are considered during the parameter tuning process.

### O3. Sensitivities to the decreasing sample sizes

**Fig. 4:**
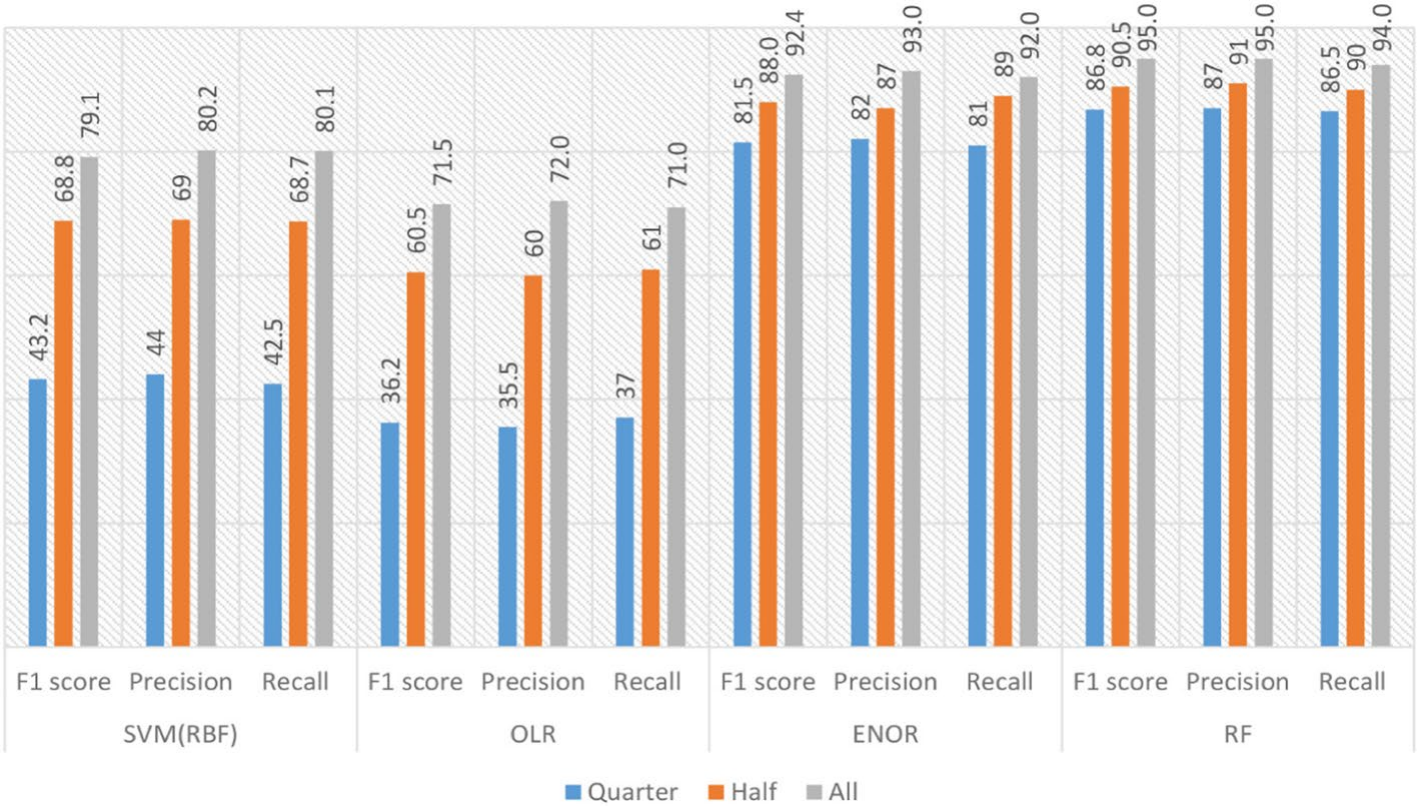
The models’ sensitivity to the size of training dataset samples

According to [76] in the case of the OLR, when the sample size is decreased, the model's classification performance is seriously affected rather than the ENOR model. However, both SVM and RF do not need a dataset that has the large number of observations, to train models with superior accuracy rates [58, 77]. Moreover, in the case of the RF, this model presents better classification performance than SVM when the training samples are less [57]. Figure 4 depicts the models' performance obtained by reducing the number of samples. As a first step, use half of the samples, and for the second attempt, only a quarter of the samples were used for training and testing the models. When these two models were trained with 50% of the observations in the samples, the observations revealed that OLR and SVM(RBF) were substantially influenced. However, the performances of the optimum RF and ENOR are not substantially influenced when these two models were trained in the same condition. The second attempt's result revealed that the optimum OLR and SVM(RBF) performances, especially the OLR's performance, were substantially influenced when these two models were trained with 25% of the observations in the samples (see Fig. 4). The weighted mean of the F1 score, Recall, and precision is approximately decreased by 14% for the SVM(RBF) and 5% for ENOR when these models are applied to half of the data. The RF’ weighted mean of F1 score, Recall, and precision decreased from 95% to 90.5%, 95% to 91%, and 94% to 90%, respectively. The OLR's weighted mean of F1 score, Recall, and precision weighted mean values decreased from 71.5% to 60.5%, 72% to 60%, and 71% to 61%. When the SVM(RBF) and ENOR models are implemented in 25% of the samples, the F1 score, Recall, and precision weighted mean values are approximately decreased by 46% and 12%, respectively. For the RF, the weighted mean F1-score value is dropped to 86.8%, precision is dropped to 87%, and Recall is dropped to 86.5% in the same condition. The selected OLR model classification performance is significantly dropped, and all three metrics values are reduced by under 38% when the model is implemented in 25% of the samples. In the case of OLR, this model's behavior is utterly compatible with the literature [76]. The previous studies revealed that, theoretically, ENOR gives better performance when it is applied to small-size training datasets [76]. In the current study, the models' behavior is compatible with the literature. In this study, due to the multicollinearity, the proposed SVM (RBF) is significantly affected when the samples are reduced. As is known, the multicollinearity is more severe in the small samples [78].

This experiment's result showed that the selected RF, and ENOR, showed better performances than the other two models when the size of training samples was reduced. As shown in Fig. 4, it can be said that there is no important change in the RF and ENOR models in this experiment's conditions in this study. Both performances of the proposed RF and ENOR models on the dataset are not substantially sensitive to the size of training samples.

### O4. Evaluation of classification performance

As shown in Table 8, in both the RF and the ENOR models, training metric results indicate that the overfitting problem did not happen. The SVM(RBF) and OLR achieved better classification performance on the training dataset. However, a weak performance is released by the models on the testing dataset. The SVM(RBF) and OLR models faced overfitting due to the multicollinearity problem in the dataset. All other proposed models reveal an accuracy of over 0.92. Moreover, the proposed RF, and ENOR models' Kappa coefficient (k) metric values, show over 0.87, in the case of the OLR, and the SVM(RBF), deliver 0.52 and 0.65, respectively. These results interpret that the OLR model's classification is in moderate agreement, the SVM (RBF) model's classification is in substantial agreement, and the classifications of other proposed models ENOR and RF, are almost in perfect agreement [79]. Nevertheless, the proposed RF model’s $k$ value is 0.90, and the weighted average of the F1-score is 0.95 which is higher than the ENOR in both metrics. The proposed models, OLR and SVM, are comparatively difficult to classify the “Inadequate” and “Adequate” classes, due to the closeness of laboratory test indicators’ reference values. According to Table 9, it is evident that all the Recall and Precision values of the “Inadequate” and “Adequate” classes in the proposed models, especially in OLR and SVM express a high probability of misclassification. However, “Deficient” class samples are fairly easy to detect and classify. Additionally, the proposed RF has a lower error rate value than the other three models.

**Table 8:** The weighted mean of SVM, OLR, ENOR, and RF classification performance on the self-collected dataset

	F1 score	Precision	Accuracy	$k$^a^	Train^b^	Recall	Specificity
SVM	0.79	0.80	0.80	0.65	0.98	0.80	0.88
OLR	0.72	0.72	0.72	0.52	0.99	0.71	0.86
ENOR	0.92	0.93	0.92	0.87	0.98	0.92	0.92
RF	0.95	0.95	0.94	0.90	0.98	0.94	0.96

^a^k term shows Cohen’ Kappa value of the models, ^b^Train shows the training accuracy

**Table 9:** The models’ confusion matrix and error rate

	Actual	Predicted			Recall	Error Rate
		Deficient	Inadequate	Adequate		
SVM	Deficient	0.86	0.06	0.08	0.86	0.20
	Inadequate	0.11	0.69	0.20	0.69	
	Adequate	0.17	0.09	0.74	0.74	
	Precision	0.90	0.77	0.55		
OLR	Deficient	0.80	0.08	0.12	0.80	0.28
	Inadequate	0.17	0.60	0.23	0.60	
	Adequate	0.13	0.26	0.61	0.61	
	Precision	0.88	0.62	0.44		
ENOR	Deficient	0.94	0.02	0.04	0.94	0.08
	Inadequate	0.03	0.91	0.06	0.91	
	Adequate	0.04	0.09	0.87	0.87	
	Precision	0.98	0.89	0.80		
RF	Deficient	0.95	0.02	0.03	0.95	
	Inadequate	0.09	0.91	0.00	0.91	0.06
	Adequate	0.00	0.04	0.96	0.96	
	Precision	0.97	0.91	0.92		

## Discussion

A comparative analysis has been performed regarding the ML models's (OLR, ENOR, SVM(RBF), and RF) sensitivities to the participant’s MtS' Positive status, hyper-parameter tuning, sensitivities to the size of training data, and the classification performance of the models. To examine the models' sensitivities and robustness, the proposed models' sensitivity to the applicants' MtS status and their robustness to the size of training dataset samples have been taken into account. The weighted mean of the F1-score, Recall, precision, sensitivity, accuracy, and Cohen’s kappa (k) has been implemented to observe the sensitivities of the proposed models.

The developed RF and ENOR are more robust than the other models when sensitivity to the applicants' MtS' Positive status has been examined. MtS predictor is one of the multicollinear features. In comparison to the OLR model, the suggested RF and ENOR are around 31% more resistant to the MtS' positive status. The data revealed that, when the test is considered in the context of the SVM(RBF), multicollinearity considerably affects the performance of the SVM(RBF). However, the performances of ENOR and the RF are not significantly affected when the MtS' status is positive. When the test was analyzed, ENOR's performance declined by 13%, while RF's performance declined by 11%. In other words, the multicollinearity problem does not have an important impact on these two models.

According to the revealed results on the samples, it can be obviously detected that RF is more robust than other models when the variations in the size of training data are examined. When the sample size is reduced for the OLR, as opposed to the ENOR model, the model's classification performance suffers significantly [76]. However, in order to train models with higher accuracy rates, SVM and RF do not require a dataset with a large number of observations [58, 77]. Additionally, when training samples are less for the RF, this model performs better than SVM in classifying data [57]. When the model is applied to 25% of the data, the performance of the chosen OLR model for classification is severely lowered, and the values of all three metrics are decreased by less than 38%. This model's behavior for OLR is completely consistent with the literature [76]. Theoretically, according to earlier work, ENOR performs better when used with limited training datasets [76]. The behavior of the models in the current investigation is consistent with the findings of the literature. The suggested SVM (RBF) in this work, however, is greatly impacted when the sample size is decreased because of the multicollinearity. As is well known, small samples have more severe multicollinearity [78] and a small sample size may make an SVM more likely to overfit in classification, which might produce false diagnostic findings [65, 66]. This experiment's result showed that the selected RF, and ENOR, showed better performances than the other two models when the size of training samples was reduced. It can be said that there is no important change in the RF and ENOR models in this experiment's conditions in this study. Both performances of the proposed RF and ENOR models on the dataset are not substantially sensitive to the size of training samples.

Alternatively, due to the proposed OLR model origin, the OLR does not need any hyper-parameters to initialize. Thus, in the current study, applying the proposed OLR model is significantly less challenging than in comparison with the other proposed models. Moreover, the proposed models' behaviors were examined when dealing with the multicollinearity problem, which was not addressed clearly in the previous study in the case of the Vitamin D level classification. The presence of the multicollinearity problem may lead the SVM(RBF) and OLR to face overfitting. Based on the origin of the OLR, overfitting is inevitable when multicollinearity exists. However, in the case of the SVM, changing the kernel function to the other multicollinearity-friendly kernel functions may overcome this challenge. The ENOR, and RF models, are not affected by the multicollinearity problem because of their model structures. These two models perform well in multiclass classification tasks on the training and testing datasets.

On the other hand, when the classification performances of the models are examined, it is clear that the RF has outperformed the SVM(RBF), ENOR, and OLR. According to training metric findings, the overfitting issue did not arise for either the RF or the ENOR models, as shown in Table 8. In terms of classification performance on the training dataset, the SVM(RBF) and OLR performed better. The SVM(RBF) and OLR models were overfitted as a result of the dataset's multicollinearity issue, which resulted in the models' poor performance on the testing dataset. The outcome is completely consistent with the findings of the earlier studies [36, 37]. All other suggested models ENOR and RF show an accuracy of more than 0.92. According to Cohen’Kappa values, the OLR model's classification is in moderate agreement, the SVM (RBF) model's classification is in substantial agreement, and the classifications of other proposed models are almost in perfect agreement [79]. Nevertheless, the proposed RF model’s k value is 0.90, and the weighted average of the F1-score is 0.95 which is higher than the ENOR in both metrics. Besides, the proposed RF’s Error rate value is less than the three other models. It is also observed from the Precision and Recall values that RF and ENOR, especially RF, classify “Inadequate” and “Adequate” classes more successfully than other models.

### Conclusions and future work

Four conventional supervised classification ML models' performance, namely: OLR, ENOR, SVM(RBF), and RF, have been compared to determine a clinically acceptable classification model for the detection of the vitamin D status of the adult participants in this study. Experiments were applied to the self-collected Cypriot adult population clinical dataset. The data collection phase was started after the NEU Scientific Ethics Committee approval and was terminated at the end of July 2022.

In conclusion, the comparative analysis revealed that the RF classifier performed better and more robust than the other proposed models in terms of accuracy (0.94), specificity (0.96), sensitivity or recall (0.94), precision (0.95), F1-score (0.95), and Cohen's kappa (0.90). Studies comparing ML algorithms conducted in the health sciences also support that RF is an efficient and superior algorithm for classification [34, 42, 45, 80]. Also in our study, it has been shown that RF is the model that gives better results in the presence of multicollinearity. For future work, the proposed RF as a suitable, and high-performance model would be further developed for the intelligent Vitamin D level detecting application with a user-friendly graphical user interface. The application could be applied for the first phase of the computer-aided system for the diagnosis of diseases caused by Vitamin D deficiency. The small sample size of the dataset is the limitation of this study; nevertheless, the authors believe that the current study could encourage organizations and scholars to apply the proposed model in a larger size sample to improve the healthcare system in Northern Cyprus.

## Data Availability

The data can be made available upon reasonable request from the corresponding author.

## Abbreviations

25-OH-D: Serum 25-Hydroxy vitamin D
Mts: Metabolic syndrome
ML: Machine learning
AI: Artificial intelligence
OLR: Ordinal logistic regression
*ENOR*: Elastic-net ordinal regression
*SVM*: Support vector machine
RF: Random forest
RBF: Radial Basis Function
NEU: Near East University
WC: Waist circumference
BMI: Body mass index
UAL: Uric acid level
SBP: Systolic blood pressure
DBP: Diastolic blood pressure
LDL: Low-density lipoprotein cholesterol
TRY: Triglycerides
HDL: High-density lipoprotein cholesterol
TCL: Total cholesterol level
hs-CRP: High-sensitivity C-reactive protein
HOMA-IR: Homeostatic model assessment–insulin resistance
FBS: Fasting blood sugar
MMS: Use of daily multivitamin and mineral supplement
FOS: Usage of daily fish oil supplement
VD: 25-OH vitamin D
VIF: Variance inflation factor
CI: Condition index
VDP: Variance decomposition proportions
